# Hearing screening using the uHear™ smartphone-based app: reproducibility of results from two response modes

**DOI:** 10.1590/2317-1782/20232021143en

**Published:** 2023-04-21

**Authors:** Marta Luiza Santana da Cunha, Márcia da Silva Lopes, Tatiane Costa Meira, Ana Paula Corona

**Affiliations:** 1 Universidade Federal da Bahia - UFBA - Salvador (BA), Brasil.; 2 Departamento de Fonoaudiologia, Universidade Federal da Bahia - UFBA - Salvador (BA), Brasil.

**Keywords:** Reproducibility of Results, Methods, Mobile Applications, Mass Screening, Hearing

## Abstract

**Purpose:**

Estimate the reproducibility of hearing screening results using the uHear™ smartphone-based app in two response modes: self-test response and test-operator.

**Methods:**

Reliability study conducted with 65 individuals aged ≥18 years assisted at the Speech-language and Hearing Therapy clinic of a public higher-education institution. Hearing screening was conducted by a single researcher using the uHear app and earbud headphones in a soundproof booth. Participants responded to sound stimuli in both self-test response mode and test-operator mode. The order in which these two uHear test modes were applied was altered according to the entrance of each participant in the study. The correspondence between the hearing thresholds obtained from each response mode was analyzed and their Intraclass Correlation Coefficient (ICC) was estimated.

**Results:**

A correspondence of ±5 dBHL >75% was observed between these hearing thresholds. The ICC values showed excellent agreement between the two response modes at all frequencies >40 dBHL tested.

**Conclusion:**

The two hearing screening response modes using the uHear app presented high reproducibility, suggesting that the test-operator mode is a viable alternative when the self-test response mode is not recommended.

## INTRODUCTION

Over the past decade, smartphone-based applications have emerged as a tool to perform hearing screening with good accuracy to identify hearing loss compared with conventional pure-tone audiometry (PTA)^(1)^. In addition, these applications are of low cost and can be self-response rapidly and easily^(1-5)^.

Studies have reinforced conventional PTA as the gold-standard examination for hearing diagnosis, but they have also pointed out that hearing screening using smartphone-based applications can be an alternative in the context of Primary Health Care (PHC) for groups at risk of hearing loss with limited access to specialized services^(1,3,6,7,8)^. Moreover, this tool can favor hearing loss assessment in large populations, contributing to both the development and implementation health prevention and promotion actions.

Several different hearing screening applications are currently available on digital platforms, with highlight for uHear™, which operates on the IOS platform and is available free of charge. This app enables assessment of 0.5-6 kHz air thresholds with maximum testing output of 90 dBHL and measure the ambient noise before the test.

Previous studies have described uHear as a promising screening tool, with sensitivity of 76-100% and specificity of 33-100%, to identify hearing loss in the self-test response mode, which is the only one provided by this application^(1,4-6,9,10)^. The test-operator mode is an alternative to record hearing screening responses that is provided by other applications, but which is not yet available on uHear.

This test mode, in which the patient’s responses to stimuli are recorded by a professional, can favor the hearing screening of individuals with motor difficulties or who are not familiar with technology. Thus, this study aimed to estimate the reproducibility of hearing screening results using the uHear app in two different response modes: self-test response mode and test-operator mode.

## METHODS

This study was approved by the Human Research Ethics Committee of the aforementioned Institution under protocol no. 2.588.097. This reliability study was conducted at the Speech-language and Hearing Therapy clinic of a public higher-education institution in northeastern Brazil between May and August 2018. Users of this clinic undergoing basic hearing assessment were invited to participate in the study. All participants signed an Informed Consent Form (ICF) prior to study commencement.

This study is part of a major project aimed at investigating the validity of hearing screening using smartphone-based applications compared with that of conventional PTA. Sample size was determined based on the results of a pilot study conducted in the major project for two different response modes using the uHear app. Initially, the means and standard deviations of the hearing thresholds generated at the tested frequencies were estimated and the lowest measures for each response mode were identified. Considering these values, those that presented a minimum difference of ±5 dBHL between the two response modes were selected. From these measures, and assuming a confidence interval of 95%, power of 80%, and ratio of 1, the study sample size was estimated in 65 individuals.

Users of the clinic aged ≥18 years were included in the study. Exclusion criteria were as follows: users with otorrhea and/or obstruction in the external acoustic meatus, or who could not understand the test procedure. All participants underwent otoscopy and answered a brief questionnaire on sociodemographic data (age, level of education, family income, and type of employment). Subsequently, the participants were instructed about the procedure for the hearing threshold screening with the assistance of prototypes of the app screen and demonstration of the different response recording modes.

Hearing screening was conducted by a single researcher in a soundproof booth using the uHear app (version 2.0.2, Unitron™, Victoria, BC, Canada) on a tablet (iPad Mini 4, Apple Inc., Cupertino, California, USA) operating on the IOS platform and earbud headphones (model CX 3.00, Sennheiser electronic, Wedemark, Hanover, Germany). The test tones at the frequencies of 1, 2, 4, 6 and 0.5 kHz were presented at 40 dBHL, initially to the right ear and then to the left ear, according to the configurations established by the manufacturer. For each frequency, the test stimulus is reduced by 10 dBHL for every positive response registered and increased by 5 dBHL when the sound is not perceived by the participant, with a hearing threshold being considered when two positive responses were recorded for every three stimuli presented^(4)^.

All participants underwent hearing screening in two different response modes: a) self-response, with the participant recording the response by touching the tablet screen upon hearing the tone presented; b) test-operator, with the researcher, positioned behind the participant, recording the response by touching the tablet screen whenever the participant raised their hand, indicating the sound detection.

The order in which the two response modes were applied on the uHear app was alternated as the participants entered the study. The hearing screening results on the uHear app are presented in a graph that identifies the degree of hearing loss. Thus, to estimate the hearing threshold numeric value, an instrument made of transparent material, prepared by the researchers, was placed on the tablet screen, allowing identification of the corresponding value. Test duration was also timed for each of the response modes used.

The data obtained were organized and analyzed on the Epidata, Epidata Analysis and Rcommander software. The differences between the hearing thresholds generated in the two response modes were calculated by subtracting the values registered in the self-response mode from those recorded in the test-operator mode. For the individuals who presented no responses, a numeric value of 95 dBHL was established as the hearing threshold, that is, 5 dBHL above the device maximum threshold. From the differences found, the frequency of correspondence between the thresholds generated in the two response modes was estimated considering variations of ≤5 dBHL and above 10 dBHL.

To identify the reproducibility of the hearing thresholds between the different response modes, the intra-rater reliability was estimated using the Intraclass Correlation Coefficient (ICC), whose values vary between 0 and 1, where ICC <0.4 indicate poor reproducibility, ICC of 0.4-0.74 represent satisfactory-to-good reproducibility, and ICC ≥0.75 correspond to excellent reproducibility^(11)^. The agreement coefficient was also calculated according to the different testing frequencies and outputs. The Student’s *t-*test was used to compare the test mean duration for the two modes.

All analyses were performed considering the responses obtained only in the right ear of each participant by drawing lots, since this study does not aim to identify the influence of the tested ear on the reproducibility of the response modes.

## RESULTS

Hearing screening using the smartphone-based uHear app was conducted with 67 participants. Two of them were excluded because of a lack of understanding of the study procedure. The 65 participants that composed study final sample were mostly women (73.8%), aged >40 years (mean=51.4; SD=16.47), and had completed high school (69.2%). Additionally, most participants reported having a family income greater than one minimum wage and not being currently employed: retired (29.2%) or housewives (15.4%).

**Figure 1:**
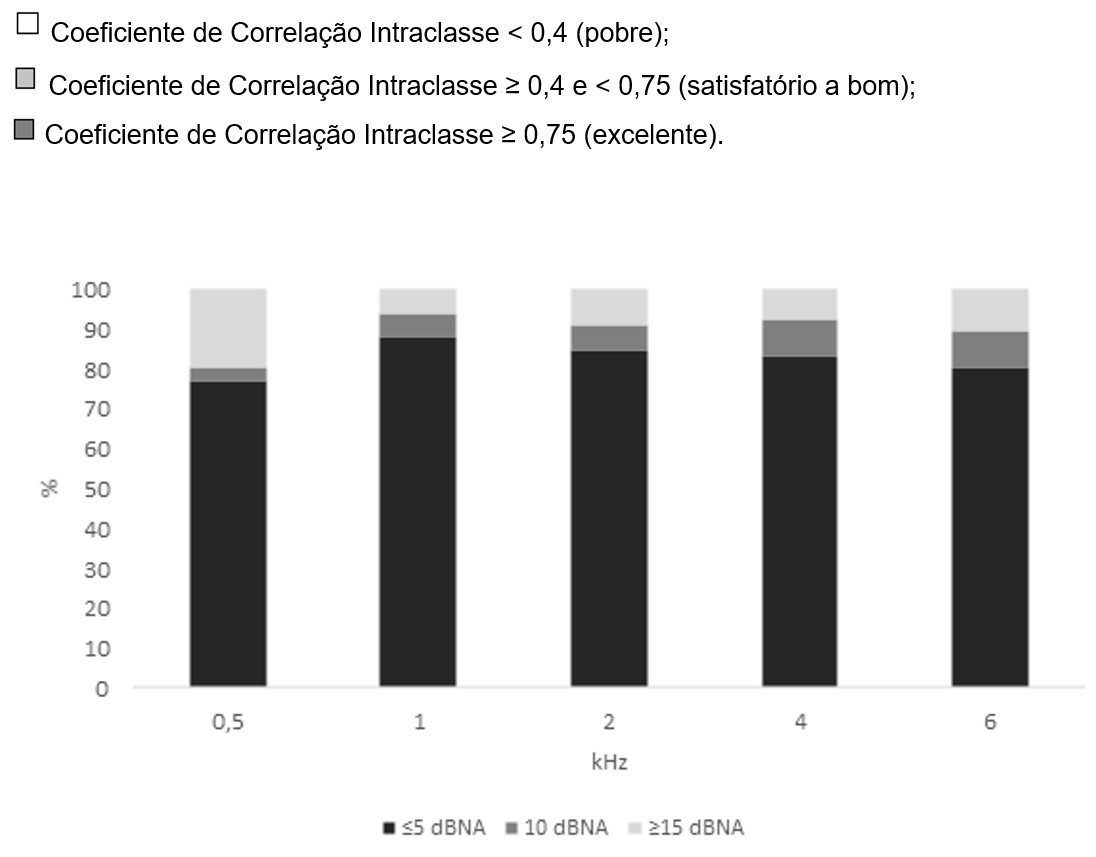
Correspondence of the hearing thresholds obtained using the self-test response and test-operator response modes by test frequency

A high correspondence (>75%) between the hearing thresholds obtained in the self-response and test-operator response modes was observed for all frequencies tested when considering differences ≤5 dBHL (Figure 1). The correspondence was >80% for differences ≤10 dBHL.

Agreement between the hearing thresholds obtained in the self-response and test-operator response modes varied between 0.826 and 0.927, indicating excellent reproducibility at all frequencies analyzed.

Table 1 shows the ICC values according to the frequency tested and the hearing threshold obtained (dBHL). All tested frequencies showed poor reproducibility between the two response modes when thresholds of 25 dBHL were analyzed. A change in the reproducibility pattern was observed for hearing thresholds of up to 30 and 60 dBHL. At the frequencies of 2,4 and 6 kHz, the ICC values showed reproducibility between satisfactory-to-good and excellent, while at the frequencies of 0.5 and 1 kHz, reproducibility was poor for thresholds ≤40 dBHL and satisfactory-to-good for thresholds from 45 to 60 dBHL. However, this pattern was not observed for thresholds ≤65 dBHL, at which reproducibility was satisfactory-to-good only at the frequencies of 2 and 4 kHz. In contrast, for hearing thresholds ≤70 and ≤95 dBHL, the reproducibility indicated by the ICC was excellent.

**Table 1:** Reproducibility between the hearing thresholds generated using the uHear app for the different response modes according to intensity and frequency (N=65)

**Frequency (Hz)**					
**Testing output (dB)**	**500**	**1000**	**2000**	**4000**	**6000**
≤15	0	*	0	0	0.186
≤20	0	*	0	0	0.186
≤25	0	0	0.270	0.288	0.277
≤30	0.123	0.122	0.552	0.584	0.458
≤35	0.197	0.172	0.662	0.642	0.493
≤40	0.359	0.374	0.733	0.797	0.545
≤45	0.468	0.437	0.508	0.839	0.685
≤50	0.553	0.597	0.798	0.869	0.798
≤55	0.597	0.643	0.838	0.885	0.812
≤60	0.654	0.643	0.852	0.893	0.830
≤65	0.165	0.140	0.439	0.422	0.205
≤70	0.692	0.765	0.872	0.831	0.871
≤75	0.692	0.807	0.891	0.873	0.871
≤80	0.758	0.859	0.919	0.873	0.871
≤85	0.778	0.908	0.919	0.885	0.871
≤90	0.778	0.917	0.931	0.896	0.744
≤95	0.826	0.927	0.873	0.908	0.831

* Insufficient number for calculation

**Caption:** Highlight in white = Intraclass Correlation Coefficient < 0.4 (Poor); Highlight in light gray = Intraclass Correlation Coefficient ≥ 0.4 and < 0.75 (Satisfactory to Good); Highlight in dark gray = Intraclass Correlation Coefficient ≥ 0.75 (Excellent)

The mean hearing assessment runtime on the uHear app in the self-test response mode was 5.63 min (SD=1.23; minimum of four and maximum of nine minutes), whereas this time was 5.55 min (SD=1.37; minimum of three and maximum of 11 minutes) in the test-operator response mode. No statistically significant difference was observed between the response modes regarding hearing screening runtime (*p*=0.65).

## DISCUSSION

The hearing thresholds obtained using the uHear app both in the self-response and test-operator response modes presented high reproducibility at all frequencies analyzed for testing outputs above 40 dBHL. This finding is evidenced both by the large occurrence of corresponding thresholds between the two response modes and by the ICC values, which ranged from satisfactory-to-good to excellent. In addition, test duration was similar between the two response modes.

This is the first investigation on hearing screening conducted in the test-operator response mode using the uHear app, thus not allowing comparison with previous results. To date, the studies conducted aimed to identify the diagnosis accuracy of this application in terms of detecting hearing loss compared with PTA or audiometry screening. In those studies, the self-response mode was used to record the responses^(1-10)^, and revealed mostly high measures of test sensitivity and specificity from this response mode. However, motor difficulties^(3)^ or lack of familiarization with touchscreen technology^(4,7)^ were reported as possible limitations to the use of this response mode, and those authors suggested that family members or caregivers assist older individuals with difficulties in handling mobile devices when conducting these tests^(3)^.

Considering that the self-test response mode can represent a barrier to individuals with difficulty or inability to use the touchscreen technology, the findings of this study point out a viable alternative mode that may favor the use of the uHear app in hearing assessments aimed at these particular populations. Likewise, the similar test runtime found for both response modes corroborates this viability.

Furthermore, the test-operator response mode favors the identification of undesirable situations at the time of assessment, such as unexpected loud ambient noise or patient fatigue, which can lead to test interruption and might not be notified by the patient in the self-test response mode.

It is also worth highlighting that hearing screening using the uHear app in the self-test response mode can accurately identify disabling hearing losses in adults (>40 dBHL in the better ear)^(1)^. Thus, the high reproducibility of the hearing thresholds observed in this study, identified at testing outputs >40 dBHL, suggests that the accuracy of the results obtained at the test-operator response mode may be similar to those observed in the self-test response mode.

In this context, it is believed that hearing assessments on uHear in the test-operator response mode can be performed by a person trained in the use of smartphones, thus enlarging its potential use in large populations, as well as in those living distant from large urban centers. Therefore, this is a tool that can be easily used in the context of PHC to identify disabling hearing loss.

Surprisingly, the findings reveal that both response modes presented low reproducibility for testing outputs ≤40 dBHL. Initially, this could be associated with the influence of ambient noise, which would hamper the detection of weak intensity tones. However, all hearing assessments were performed in a soundproof booth, thus minimizing this influence.

Another plausible explanation for this result is the effect of learning, since hearing screening in the two different response modes was carried out in sequence. Although the first response mode was alternated among the participants, which could have reduced the effect of learning on a specific response mode, it should not be discarded the possibility that this effect may have favored better thresholds in the second testing, regardless of the response mode used.

Moreover, among individuals with hearing close to the normality patterns (<40 dBHL), it is believed that their participation in this study may have been their first contact with the detection of pure tones, as well as with a hearing assessment procedure. Thus, the hearing thresholds generated from the first response mode tested may have been worse compared with those of participants with previous experience in hearing assessments.

It is also important to highlight, as a potential limitation to this study, that the participants were selected in a hearing assessment service, where there are a large number of individuals with hearing loss, which compromised the reproducibility analysis for weak intensity tones.

It should also be considered the potential influence of lack of familiarization of some individuals with the touchscreen technology when obtaining hearing thresholds in the self-test response mode. To minimize this potential bias, all participants were introduced to prototypes of the initial and final screens of the application, and a detailed explanation on how and where they should register their responses on the smartphone screen was provided.

In contrast, the influence of the participation of a researcher in the process of recording the responses in the test-operator mode also should not be discarded, since the uHear app automatically manages the interval between the stimulus presentations as well as the time provided to record the responses. Thus, the worse results may have been obtained because of the increase in the time elapsed between signaling the test tone detection by the patient and recording this response on the smartphone screen by the researcher.

## CONCLUSION

Preliminary evidence of this study showed high reproducibility for the two response modes to the hearing screening using the uHear app. Thus, in addition to the self-test response mode, suggested by the application developer, the test-operator response mode can also be used in hearing screening to identify disabling hearing losses, thus enabling the assessment of individuals with motor difficulties or those who are not familiar with the touchscreen technology.
