# Supplementary material for: Hearing screening using the uHear™ smartphone-based app: reproducibility of results from two response modes
Source: Codas. 2023 Apr 21;35(2):e20210143. doi: 10.1590/2317-1782/20232021143en (PMC10137764; doi:10.1590/2317-1782/20232021143en)
Supplement: Translation

# Translation: Triagem auditiva com o aplicativo para dispositivos móveis uHear™: reprodutibilidade dos resultados utilizando dois diferentes modos de resposta

**DOI:** 10.1590/2317-1782/20232021143pt

**Authors:** Marta Luiza Santana da Cunha, Márcia da Silva Lopes, Tatiane Costa Meira, Ana Paula Corona

**Affiliations:** 1 Universidade Federal da Bahia - UFBA - Salvador (BA), Brasil.; 2 Departamento de Fonoaudiologia, Universidade Federal da Bahia - UFBA - Salvador (BA), Brasil.

**Keywords:** Reprodutibilidade dos Testes, Métodos, Aplicativos Móveis, Programas de Rastreamento, Audição

## Abstract

**Objetivo:**

Estimar a reprodutibilidade dos resultados da triagem auditiva com o aplicativo uHear, utilizando dois diferentes modos de resposta, o modo autoaplicado e o modo com intermediação do pesquisador.

**Método:**

Estudo de confiabilidade realizado com 65 indivíduos, maiores de 18 anos, que compareceram à clínica escola de Fonoaudiologia de uma instituição pública de ensino superior. A triagem auditiva foi realizada com o aplicativo uHear, em cabina acústica, utilizando fone intra-auricular e conduzida sempre pelo mesmo pesquisador. Os participantes responderam ao estímulo sonoro de maneira autoaplicada e com intermediação do pesquisador. A ordem de realização dos dois modos de resposta ao aplicativo uHear foi alternada de acordo com a entrada do participante no estudo. Foi analisada a correspondência entre os limiares auditivos obtidos com os dois modos de resposta, bem como estimado o Coeficiente de Correlação Intraclasse (ICC).

**Resultados:**

Verificou-se uma correspondência de ± 5 dBNA superior a 75% entre os limiares auditivos obtidos nos dois modos de resposta. O ICC revelou concordância excelente entre os dois modos de resposta, em todas as frequências testadas, para intensidades superiores a 40 dBNA.

**Conclusão:**

Os dois modos de resposta à triagem auditiva com o aplicativo uHear apresentam elevada reprodutibilidade, o que permite indicar o modo de resposta com intermediação do pesquisador como uma alternativa viável quando o modo autoaplicado não for recomendado.

## INTRODUÇÃO

Na última década, aplicativos para dispositivos móveis surgem como uma ferramenta para realização de triagens auditivas com boa acurácia para identificação da perda auditiva, em comparação com a audiometria convencional^(1)^. Adicionalmente, são de fácil e rápida aplicação, baixo custo e podem ser utilizados de maneira autoaplicada^(1-5)^.

Embora as investigações já conduzidas reforcem que audiometria convencional é o exame padrão-ouro para o diagnóstico auditivo, também apontam que a triagem auditiva com aplicativos para dispositivos móveis pode ser uma alternativa, a ser utilizada em Unidades Básicas de Saúde, para a promoção do cuidado em grupos populacionais de risco para a perda auditiva, com acesso limitado a serviços especializados de saúde auditiva^(1,3,6,7,8)^. Além disso, esta ferramenta pode favorecer a estimativa da perda auditiva em grandes populações, colaborando para a elaboração e implementação de ações de prevenção e promoção de saúde.

Diferentes aplicativos para triagem auditiva são atualmente disponibilizados nas plataformas digitais. Dentre eles, destaca-se o uHear, o qual opera na plataforma IOS e é disponibilizado gratuitamente. Adicionalmente, este aplicativo permite avaliar os limiares aéreos de 0,5 a 6 kHz ofertando tons com intensidade máxima de 90 dBNA e possibilita a medição do nível de ruído ambiente^(1)^.

Estudos prévios indicam o uHear como uma ferramenta promissora de triagem, com sensibilidade de 76% a 100% e especificidade de 33% a 100%, para a identificação da perda auditiva, utilizando o modo de resposta autoaplicado, o qual é disponibilizado pelo aplicativo^(1,4-6,9,10)^. O modo de resposta com intermediação do examinador é uma alternativa para o registro de respostas à triagem auditiva, ofertada por outros aplicativos, mas ainda não disponível no uHear.

Este modo de resposta, no qual o registro da resposta no dispositivo é realizado por um examinador sempre que o indivíduo informa a detecção do tom teste, pode favorecer a triagem auditiva de indivíduos que não têm aproximação com a tecnologia ou pessoas com dificuldades motoras. Assim, o objetivo deste estudo foi estimar a reprodutibilidade dos resultados da triagem auditiva com o aplicativo uHear, utilizando dois diferentes modos de resposta, o modo autoaplicado e o modo com intermediação.

## MÉTODO

Este estudo foi aprovado pelo Comitê de Ética em Pesquisa com Seres Humanos da instituição proponente sob protocolo número 2.588.097. Trata-se de um estudo de confiabilidade realizado, no período de maio a agosto de 2018, em uma clínica escola de Fonoaudiologia de uma instituição pública de ensino superior da região nordeste do país. Foram convidados a participar do estudo os usuários que compareceram à referida clínica para realização de avaliação audiológica básica e todos que concordaram assinaram o Termo de Consentimento Livre e Esclarecido.

Este estudo está vinculado a um Projeto mãe, o qual tem por objetivo investigar a validade da triagem auditiva com aplicativos para smartphone em comparação com a audiometria tonal liminar. O tamanho da amostra do presente estudo foi determinado a partir dos resultados do estudo piloto do Projeto mãe, utilizando-se os dois diferentes modos de resposta na triagem auditiva com o aplicativo uHear. Inicialmente, foram estimadas as médias e desvios padrões dos limiares auditivos obtidos nas frequências testadas e identificadas as menores medidas para cada um dos modos de resposta. Considerando estes valores, foram selecionados aqueles que apresentavam uma diferença mínima de 5 dB entre os dois modos de resposta. A partir destas medidas, assumindo um intervalo de confiança de 95%, poder de 80% e razão 1, foi estimada a participação de 65 indivíduos no estudo.

Foram incluídos os usuários com idade superior a 18 anos, e excluídos aqueles que apresentavam otorréia, obstrução em meato acústico externo ou que não compreenderam o procedimento do teste. Todos os participantes foram submetidos à meatoscopia e responderam a um breve questionário que abordou dados sociodemográficos (idade, escolaridade, renda e inserção no mercado de trabalho). Em seguida foi realizada orientação sobre o procedimento de pesquisa dos limiares auditivos, com o auxílio de protótipos da tela do aplicativo, e demonstração dos diferentes modos de registro das respostas.

A triagem auditiva foi realizada sempre pelo mesmo pesquisador, em cabina acústica, utilizando o aplicativo para smartphone uHear (versão 2.0.2, Unitron^™^, Victoria, BC, Canadá) através de tablet com sistema operacional IOS (iPad Mini 4, Apple Inc., Cupertino, California, EUA) e fones de ouvido intra-aurais (modelo CX.3.00, Sennheiser electronic, Wedemark, Hanôver, Alemanha). Os tons teste nas frequências de 1, 2, 4, 6 e 0,5 kHz foram apresentados em 40 dBNA, inicialmente na orelha direita e posteriormente na orelha esquerda, conforme as configurações estabelecidas pelo fabricante do aplicativo. Para cada frequência, o estímulo teste é reduzido em 10 dBNA a cada resposta positiva registrada e aumentado em 5 dBNA quando o som não foi percebido pelo participante, sendo considerado limiar auditivo quando registradas duas respostas positivas para três estímulos apresentados^(4)^.

Todos os participantes realizaram a triagem auditiva com dois diferentes modos de resposta: a) autoaplicado, no qual o próprio participante registrou a resposta com o toque na tela do tablet sempre que ouvia o som apresentado; b) com intermediação, no qual o pesquisador, posicionado atrás do participante, registrava a resposta tocando na tela do tablet sempre que o participante levantava a mão, indicando a detecção do som.

A ordem de realização dos dois modos de resposta ao aplicativo uHear foi alternada de acordo com a entrada do participante no estudo. Os resultados da triagem auditiva com o uHear são apresentados em forma de gráfico, identificando o grau da perda auditiva. Assim, para a estimativa do valor numérico do limiar auditivo foi utilizado um instrumento em material transparente, elaborado pelos pesquisadores, que ao ser sobreposto a tela do tablet permitiu a identificação do valor correspondente. Também foi registrado o tempo de execução do teste em cada um dos modos de resposta.

Os dados obtidos foram organizados e analisados nos programas Epidata, Epidata Analysis e Rcommander. Foram calculadas as diferenças entre os limiares auditivos obtidos nos dois modos de resposta mediante a subtração dos valores dos limiares identificados através do modo autoaplicado e dos valores dos limiares registrados no modo com intermediação do pesquisador. Para os indivíduos que apresentaram ausência de respostas foi estabelecido o valor numérico de 95 dBNA como limiar auditivo, isto é, 5 dBNA acima do limite máximo do equipamento. A partir das diferenças encontradas foi estimada a frequência de correspondência entre os limiares obtidos nos dois modos de resposta, considerando variações de até 5 dBNA, iguais a 10 dBNA e superiores a 10 dBNA.

Para a identificação da reprodutibilidade dos limiares auditivos entre os diferentes modos de resposta, foi analisada a concordância do teste intraobservador por meio do Coeficiente de Correlação Intraclasse (CCI), o qual varia entre 0 e 1. Valores de CCI inferiores a 0,4 indicam reprodutibilidade pobre. Já a reprodutibilidade satisfatória a boa é identificada quando o CCI varia de 0,4 a 0,74 e valores iguais e superiores a 0,75 revelam que a reprodutibilidade é excelente^(11)^. O coeficiente de concordância também foi calculado de acordo com as diferentes frequências e intensidades. O teste *t* de *Student* foi utilizado para comparar as médias do tempo de execução do teste nos dois modos.

Todas as análises do presente estudo foram realizadas considerando as respostas obtidas apenas na orelha direita de cada participante, estabelecida mediante sorteio. Esta decisão metodológica foi baseada no fato de que o estudo não tem o propósito de identificar a influência da orelha testada na reprodutibilidade dos modos de resposta.

## RESULTADOS

A triagem auditiva com o aplicativo para smartphone uHear foi realizada com 67 participantes, sendo dois excluídos por não compreensão do procedimento do estudo. Entre os 65 participantes incluídos na pesquisa, a maioria era do gênero feminino (73,8%), apresentava faixa etária superior a 40 anos, com média de idade de 51,4 anos (DP=16,47) e escolaridade igual ou superior ao ensino médio (69,2%). Adicionalmente, a maioria dos participantes relatou renda familiar superior a um salário-mínimo e não inserção no mercado de trabalho, declarando-se como aposentado (29,2%) e dona de casa (15,4%).

Observou-se uma elevada correspondência (> 75%) entre os limiares obtidos no modo de resposta autoaplicado e com intermediação do pesquisador, para todas as frequências testadas, quando consideradas diferenças de até 5 dBNA (Figura 1). Os percentuais de correspondência foram superiores a 80% para diferenças de até 10 dBNA.

**Figura 1:** Correspondência dos limiares auditivos obtidos no modo autoaplicado e com intermediação do pesquisador, por frequência de teste

A concordância entre os limiares auditivos obtidos nos modos autoaplicado e com intermediação do pesquisador variou de 0,826 a 0,927 indicando uma reprodutibilidade excelente em todas as frequências pesquisadas.

Na Tabela 1 são apresentados os CCIs de acordo com a frequência testada e o limiar auditivo obtido em dBNA. Para todas as frequências testadas, verificou-se que a reprodutibilidade entre os dois modos de resposta é pobre quando analisados limiares de 25 dBNA. Observa-se uma mudança no padrão de reprodutibilidade para limiares auditivos de até 30 e 60 dBNA. Nas frequências de 2, 4 e 6 kHz verificou-se que os valores do CCI apresentaram uma reprodutibilidade satisfatória a excelente, enquanto nas frequências de 0,5 e 1 kHz a reprodutibilidade foi pobre para limiares até 40 dBNA e satisfatória a boa para limiares de 45 a 60 dBNA. Entretanto, este padrão não foi observado para limiares até 65 dBNA, nos quais a reprodutibilidade foi satisfatória a boa apenas nas frequências de 2 e 4 kHz. Por outro lado, para limiares auditivos até 70 e 95 dBNA a reprodutibilidade indicada pelo CCI foi excelente.

**Tabela 1:** Reprodutibilidade entre os limiares auditivos obtidos com o uHear nos diferentes modos de resposta, de acordo com a intensidade e frequência (N = 65)

Frequências (Hz)					
Intensidade (dB)	**500**	**1000**	**2000**	**4000**	6000
≤ 15	0	*	0	0	0,186
≤ 20	0	*	0	0	0,186
≤ 25	0	0	0,270	0,288	0,277
≤ 30	0,123	0,122	0,552	0,584	0,458
≤ 35	0,197	0,172	0,662	0,642	0,493
≤ 40	0,359	0,374	0,733	0,797	0,545
≤ 45	0,468	0,437	0,508	0,839	0,685
≤ 50	0,553	0,597	0,798	0,869	0,798
≤ 55	0,597	0,643	0,838	0,885	0,812
≤ 60	0,654	0,643	0,852	0,893	0,830
≤ 65	0,165	0,140	0,439	0,422	0,205
≤ 70	0,692	0,765	0,872	0,831	0,871
≤ 75	0,692	0,807	0,891	0,873	0,871
≤ 80	0,758	0,859	0,919	0,873	0,871
≤ 85	0,778	0,908	0,919	0,885	0,871
≤ 90	0,778	0,917	0,931	0,896	0,744
≤ 95	0,826	0,927	0,873	0,908	0,831

* Número insuficiente para a realização do cálculo

**Legenda:** Destaque em branco = Coeficiente de Correlação Intraclasse < 0,4 (Pobre); Destaque em cinza claro = Coeficiente de Correlação Intraclasse ≥ 0,4 e < 0,75 (Satisfatório a Bom); Destaque em cinza escuro = Coeficiente de Correlação Intraclasse ≥ 0,75 (Excelente)

O tempo médio de execução do aplicativo uHear no modo de resposta autoaplicado foi 5,63 minutos (DP = 1,23; mínimo de quatro e máximo de nove). Já no modo com intermediação do pesquisador o tempo médio foi 5,55 minutos (DP = 1,37; mínimo três e máximo onze). Não foi observada diferença estatisticamente significante entre os modos de resposta, quanto ao tempo de execução (p=0,65).

## DISCUSSÃO

Os limiares auditivos obtidos com o aplicativo uHear através do modo de resposta com intermediação do pesquisador e do modo autoaplicado apresentam elevada reprodutibilidade, em todas as frequências pesquisadas, para intensidades superiores a 40 dBNA. Este achado é evidenciado tanto pela elevada ocorrência de limiares correspondentes entre os dois modos de resposta, quanto por coeficientes de correlação intraclasse de satisfatório a bom e excelentes. Além disso, o tempo de execução do teste nos dois modos de resposta foi semelhante.

O presente estudo foi a primeira investigação conduzida utilizando o modo de resposta com intermediação do pesquisador para a triagem auditiva com o aplicativo uHear, o que não permite comparações com resultados prévios. Até o momento, as pesquisas conduzidas tiveram por objetivo identificar a acurácia diagnóstica do aplicativo para identificação da perda auditiva, em comparação com a audiometria tonal liminar ou triagem audiométrica. Nestas pesquisas o modo autoaplicado foi utilizado para o registro das respostas^(1-10)^ e, em sua maioria, revelam elevadas medidas de sensibilidade e especificidade do teste com este modo de resposta. Contudo, dificuldades motoras^(3)^ ou não proximidade com a tecnologia *touch screen* ^(4,7)^ foram apresentadas como possíveis limitações para o uso deste modo de resposta, sendo sugerido o auxílio de familiares ou cuidadores para o uso de testes com aplicativos em idosos com dificuldades no manuseio de dispositivos móveis^(3)^.

Considerando que o modo autoaplicado pode representar uma barreira para os indivíduos que apresentam dificuldade ou incapacidade com o uso da tecnologia *touch screen*, os achados do presente estudo apontam uma alternativa viável de modo de resposta que pode favorecer a utilização do aplicativo em investigações auditivas nas populações com tal particularidade. Da mesma forma, o tempo de execução similar para ambos os modos de resposta corrobora essa viabilidade.

Adicionalmente, o modo de registro intermediado por um pesquisador/examinador favorece a identificação de situações não desejáveis para o momento da avaliação, como elevados ruídos ambientais inesperados ou demonstrações de fadiga por parte do examinado, as quais exigiriam a interrupção do teste, mas poderiam não ser notificadas pelo examinado no modo de resposta autoaplicado.

Cabe ressaltar ainda que a triagem auditiva com o aplicativo uHear, utilizando o modo de resposta autoaplicado, apresenta boa acurácia para a identificação de perdas auditivas incapacitantes em adultos (> 40 dBNA na melhor orelha)^(1)^. Assim, a elevada reprodutibilidade dos limiares auditivos no presente estudo, identificada em intensidades superiores a 40 dBNA, permite inferir que a acurácia dos testes realizados com o modo intermediado por um pesquisador/examinador pode ser semelhante àquela observada no modo autoaplicado.

A partir deste cenário, acredita-se que a avaliação auditiva com uHear, utilizando o modo de resposta com intermediação do pesquisador, pode ser mediada por pessoa treinada para manusear o smartphone, ampliando o seu potencial de utilização para triagens auditivas de grandes populações, bem como de populações distantes de grandes centros urbanos, se configurando como uma ferramenta de fácil aplicabilidade em ações na rede básica de atenção à saúde para identificação da perda auditiva incapacitante.

Surpreendentemente, nossos achados revelam que para intensidades até 40 dBNA a reprodutibilidade dos modos de resposta foi baixa. Inicialmente é possível relacionar este achado com a influência do ruído ambiental, o qual dificultaria a detecção de tons teste apresentados em fraca intensidade. Contudo, todas as avaliações auditivas do presente estudo foram realizadas em cabina acústica, minimizando, assim, esta influência.

Outra explicação plausível para este resultado seria o efeito da aprendizagem, já que a triagem auditiva através dos dois diferentes modos de resposta ao teste foi realizada em sequência. Apesar de alternarmos o primeiro modo de resposta ao teste entre os participantes, o que potencialmente minimizaria o efeito de aprendizagem para um modo de resposta específico, não é possível descartar que este efeito tenha favorecido melhores limiares na segunda testagem, independentemente do modo de resposta utilizado.

Adicionalmente, entre indivíduos com audição próxima aos padrões de normalidade (< 40 dBNA), acredita-se que a participação no presente estudo pode ter sido o primeiro contato com a detecção de tons puros, bem como com o procedimento de avaliação auditiva. Assim, é possível que os limiares auditivos obtidos no primeiro modo de resposta testado tenham sido piores em comparação com aqueles participantes com experiência prévia de avaliação auditiva.

É importante destacar ainda, como potencial limitação do presente estudo, a seleção dos participantes a partir de um serviço de avaliação audiológica, incluindo maior número de indivíduos com perda auditiva, o que comprometeu a análise da reprodutibilidade para tons de fraca intensidade.

Deve-se considerar também a possível influência na obtenção dos limiares auditivos no modo de resposta autoaplicado em indivíduos que não tenham proximidade com a tecnologia *touch screen*. Para minimizar este possível viés foi apresentado a todos os participantes protótipos da tela inicial e final do aplicativo e explicação detalhada de como e onde os participantes deveriam registrar suas respostas na tela do dispositivo móvel.

Em contraponto, não podemos descartar a influência da inclusão do pesquisador no processo de registro das respostas no modo com intermediação, pois o aplicativo uHear gerencia automaticamente o intervalo entre as apresentações dos estímulos, bem como o tempo destinado para o registro da resposta. Assim, é possível que piores resultados tenham sido obtidos em função do aumento do tempo decorrido entre a sinalização da detecção do tom teste, pelo examinado, e o registro desta resposta na tela do dispositivo móvel, pelo pesquisador.

## CONCLUSÃO

As evidências preliminares deste estudo demonstraram que os dois modos de resposta à triagem auditiva com o aplicativo uHear apresentam elevada reprodutibilidade. Assim, além do modo de resposta autoaplicado, sugerido pelo desenvolvedor do aplicativo, o modo com intermediação do pesquisador também pode ser utilizado na triagem auditiva para identificação de perdas auditivas incapacitantes, viabilizando a avaliação de indivíduos com dificuldades motoras ou que não tenham aproximação com a tecnologia *touch screen*.
